# Progression-free survival exceeding four years after transarterial embolization for liver metastases from triple-negative breast cancer: a case report

**DOI:** 10.3389/fonc.2026.1804977

**Published:** 2026-04-16

**Authors:** Shengjie Liu, Mirinisa Kaicaier, Aisaide Aikelaimu, Yuhan Zhang, Xueping Hou, Yuying Wang, Liyuan Ma, Weihua Jiang

**Affiliations:** The Second Department of Breast Surgery, The Affiliated Tumor Hospital of Xinjiang Medical University, Xinjiang Uygur Autonomous Region, Urumqi, China

**Keywords:** interventional radiology, liver metastases, oligometastatic, progression-free survival, transarterial embolization (TAE), triple-negative breast cancer (TNBC)

## Abstract

Liver metastasis in triple-negative breast cancer (TNBC) is associated with an unfavorable prognosis. Prior studies have reported a median overall survival of approximately 3–15 months after the diagnosis of liver metastasis, with a 5-year survival rate of less than 12%. We present the case of a 46-year-old woman who developed a solitary liver metastasis 14 months following curative-intent surgery for triple-negative breast cancer (TNBC). Following multidisciplinary team (MDT) evaluation, she received superselective hepatic arterial transarterial embolization (TAE) in combination with a TP regimen (paclitaxel and carboplatin). After a partial response was achieved per Response Evaluation Criteria in Solid Tumors (RECIST) 1.1, therapy was de-escalated to one year of oral capecitabine maintenance. Following multimodal therapy, the liver metastasis demonstrated substantial radiologic regression and durable disease control. The patient has remained progression-free for more than 48 months (PFS > 4 years), an outcome that is uncommon in metastatic TNBC. This case supports the concept that, in carefully selected patients with solitary TNBC liver metastasis, combining locoregional interventional therapy with systemic treatment may yield prolonged tumor control. By reducing intrahepatic tumor burden, locoregional embolization (e.g., TAE) may extend the effective treatment window for systemic therapy and may contribute to improved long-term outcomes.

## 1 Introduction

Compared to other breast cancer subtypes, patients with triple-negative breast cancer (TNBC) have a higher rate of distant recurrence and a poorer prognosis (1). The risk of early distant recurrence within five years of diagnosis is approximately three times that of other subtypes (1). Due to the absence of target receptors—namely estrogen receptor, progesterone receptor, and human epidermal growth factor receptor 2 (HER2)—treatment options are limited (2). It has been reported that in about 13.7% of TNBC cases, liver metastasis is the initial site of recurrence (3). While liver metastasis from breast cancer is typically regarded as a manifestation of systemic disease with a poor prognosis, hepatic arterial embolization (TAE)has emerged as a relatively safe treatment option that shows promise in certain patients, potentially prolonging progression-free survival (PFS).Locoregional transarterial therapies, including TAE, transarterial chemoembolization (TACE), and transarterial radioembolization (TARE), have been increasingly applied across various solid tumors with liver-dominant disease (4).However, published data on transarterial interventions for breast cancer liver metastases (BCLM) predominantly involve mixed molecular subtypes, and reports specifically addressing TNBC are exceedingly rare. Systematic reviews have reported median overall survival of 15.3 months for TACE and 11.9 months for TARE in BCLM (5), with median disease-free survival ranging from 2.9 to 17.0 months (6). A meta-analysis (7) pooling 26 studies (1,266 patients) found pooled median over survival(OS) of 17.8 months for TACE and 9.2 months for TARE. To our knowledge, PFS exceeding four years following bland TAE specifically in TNBC liver metastasis has not been previously documented. In this report, we present a case of a TNBC patient with liver metastasis who achieved a PFS of over four years and complete remission following TAE treatment.

## 2 Case presentation

### 2.1 Initial diagnosis and clinical course

In March 2020, a 46-year-old premenopausal woman presented with a 1-month history of a palpable right breast lump. On examination, the breasts were symmetric and well developed. The right nipple was inverted, without nipple–areolar erosion. No skin dimpling, peau d’orange, erythema, or ulceration was noted. A firm mass measuring approximately 3.0 × 2.0 cm was palpated in the upper inner quadrant of the right breast, located ~1.0 cm from the nipple. The lesion demonstrated limited mobility, with no evidence of overlying skin involvement and no fixation to the chest wall. There was no nipple discharge. No palpable supraclavicular or axillary lymphadenopathy was detected bilaterally.

**Figure 1:**
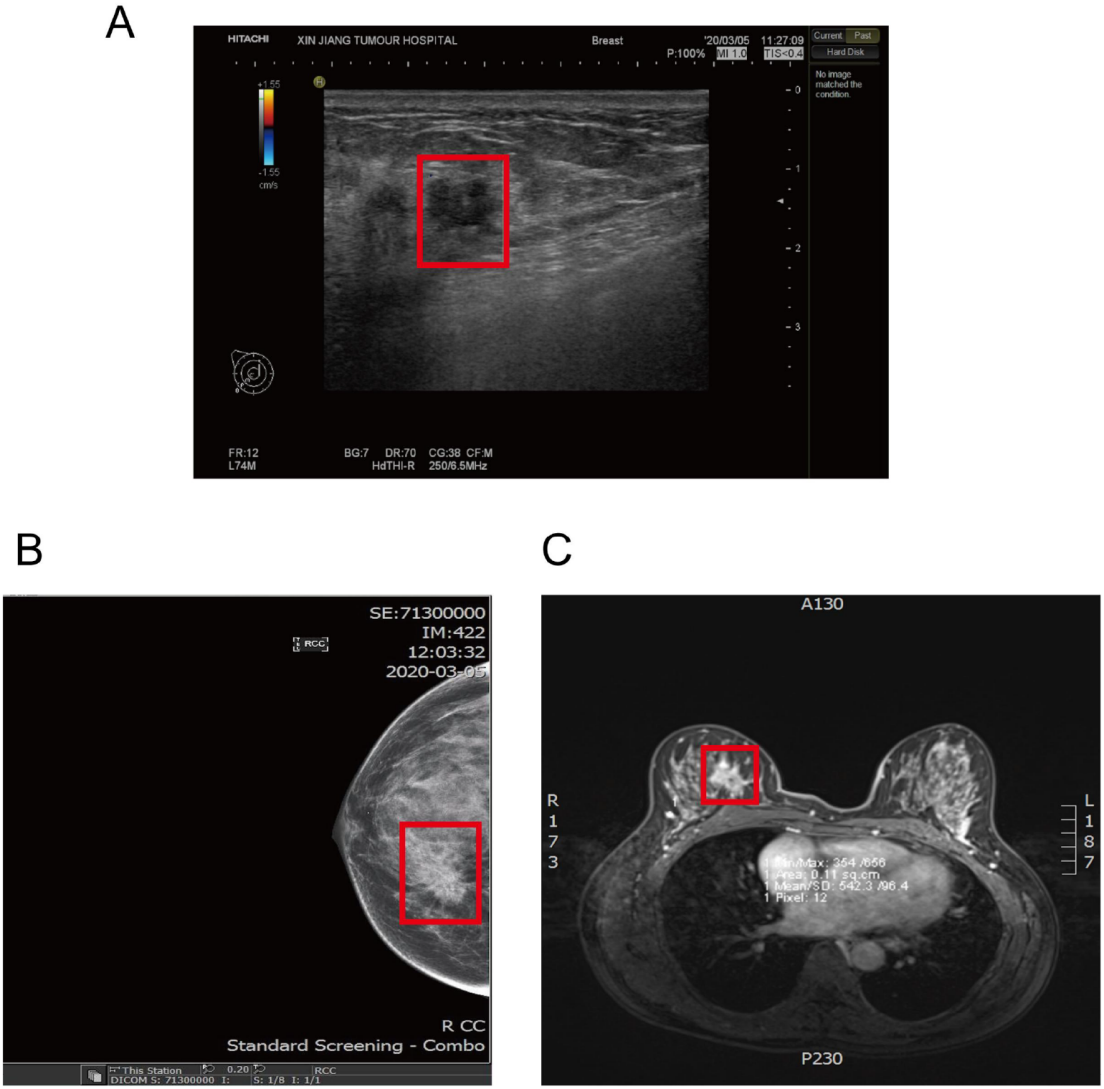
Initial diagnostic. **(A)** Color Doppler ultrasonography (March 5, 2020) showed an irregular solid hypoechoic mass in the upper inner quadrant of the right breast (3.3 × 2.4 × 2.3 cm, inside the red box), with indistinct margins and intralesional vascular signals. **(B)** Mammography (March 5, 2020) demonstrated an irregular high-density mass in the upper inner quadrant of the right breast (approximately 27 × 15 mm, inside the red box) with spiculated margins; nipple inversion was noted without obvious skin thickening. **(C)** Breast MRI (March 9, 2020)revealed an irregular spiculated mass in the upper inner quadrant (27 × 19 × 23.6 mm, inside the red box) with diffusion restriction and heterogeneous rim enhancement with central non-enhancing areas; the time–intensity curve showed an early enhancement–plateau (type II) pattern.

Diagnostic work-up: Breast ultrasound identified a solid mass in the upper inner quadrant of the right breast (BI-RADS 4C), suspicious for malignancy (**Figure 1A**). Mammography demonstrated a right breast mass categorized as BI-RADS 5 (highly suggestive of malignancy) (**Figure 1B**). Breast MRI further confirmed a lesion in the upper inner quadrant of the right breast and revealed multiple enlarged right axillary lymph nodes (**Figure 1C**). Additionally, no metastatic lesions were detected in the patient’s whole-body imaging studies following admission.

Ultrasound-guided core needle biopsy of the breast lesion confirmed invasive ductal carcinoma, histologic grade II. Immunohistochemistry showed estrogen receptor (ER) negative, progesterone receptor (PR) negative, HER2 1+ (IHC), and a high proliferative index (Ki-67 ~90%). Taken together, the clinicoradiologic and pathologic findings supported a diagnosis of right-sided triple-negative breast cancer (TNBC).

On March 11, 2020, the patient underwent right breast-conserving surgery with sentinel lymph node biopsy. Final pathology confirmed invasive breast carcinoma of no special type (NST), histologic grade III (Nottingham score 8), with a maximal tumor size of 1.5 cm. Immunohistochemistry showed ER negative, PR negative, HER2 1+ (IHC), and Ki-67 approximately 50%; AR was 1+ in ~30% of tumor cells. Additional markers included CK5/6 positive, p63 negative, and GATA3 positive; p53 showed a null pattern. Sentinel lymph node metastasis was present in 2 of 6 nodes examined. The disease was staged as pT1N1(sn)M0 (stage IIA). Given the triple-negative phenotype and nodal involvement, adjuvant chemotherapy and radiotherapy were indicated. In accordance with CSCO breast cancer guidelines, an anthracycline- and taxane-based regimen was recommended; therefore, an 8-cycle AC-T regimen (anthracycline plus cyclophosphamide followed by a taxane) was planned for this patient.

From September 16, 2020 to October 23, 2020, the patient received intensity-modulated radiotherapy (IMRT) to the right breast, right supraclavicular region, and right axilla with a dose of 5040 cGy in 28 fractions, with a simultaneous boost to the tumor bed to 6020 cGy in 28 fractions. During radiotherapy, the patient developed grade III myelosuppression, and mild skin reactions were observed within the irradiated field.

### 2.2 Recurrence and diagnosis of liver metastasis

**Figure 2:**
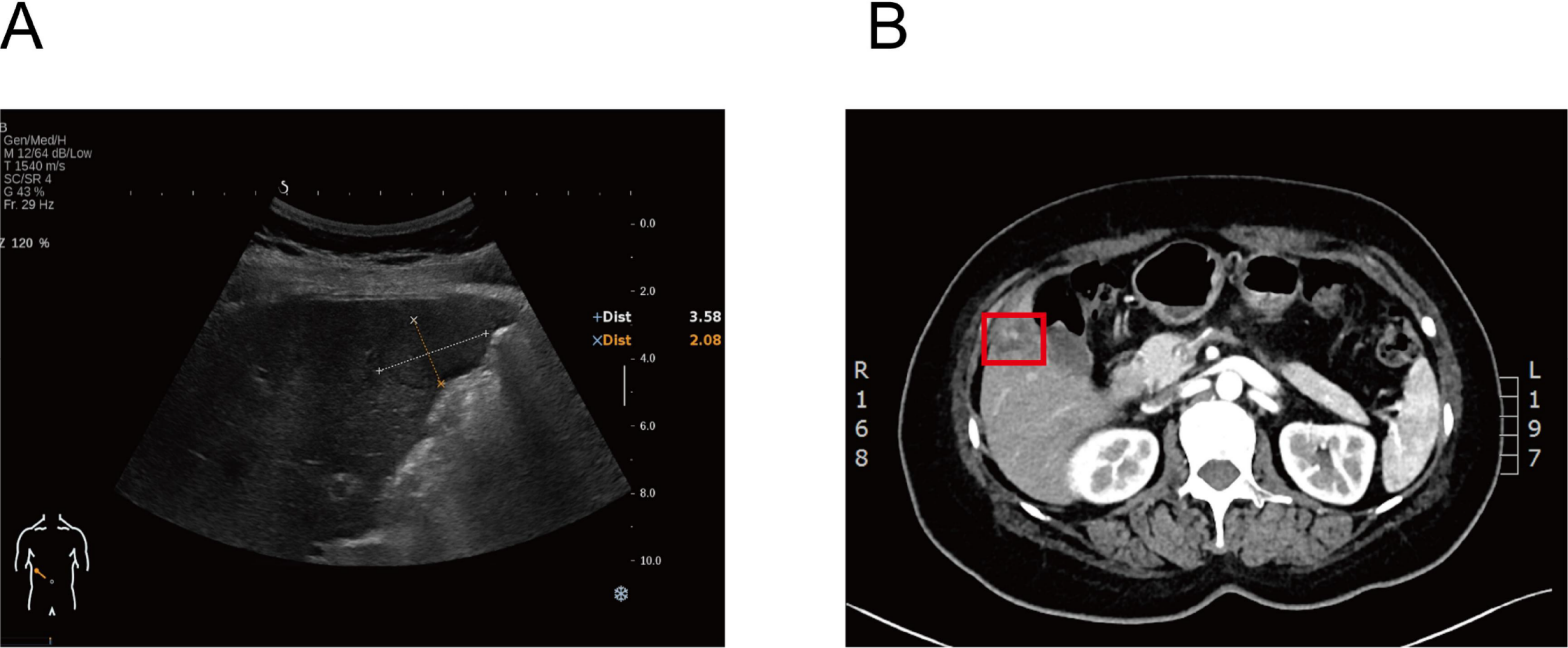
Metastatic imaging. **(A)** Ultrasound (May 2, 2021): The liver was grossly normal in size and contour. A well-circumscribed solid hypoechoic nodule (~3.5 × 2.1 cm) was identified near the gallbladder fossa with heterogeneous internal echotexture. **(B)** Contrast-enhanced CT (May 2, 2021): CT demonstrated a hypodense lesion in hepatic segment V (~3.0 × 2.7 cm, inside the red box) with prominent rim enhancement and central necrosis, associated with focal capsular bulging.

The patient’s Eastern Cooperative Oncology Group (ECOG) performance status was 1. Laboratory evaluation revealed elevated transaminases (ALT 96 U/L, AST 148 U/L) with normal bilirubin levels. The patient underwent routine surveillance every 3–6 months after surgery. Fourteen months postoperatively, abdominal ultrasonography detected a solid intrahepatic lesion suspicious for metastasis (**Figure 2A**). Subsequent inpatient staging/work-up revealed no evidence of extrahepatic metastases. Contrast-enhanced abdominal CT confirmed a mass in hepatic segment V, consistent with a metastatic lesion (**Figure 2B**).

Given the superficial location of the segment V (S5) hepatic lesion—adjacent to the liver capsule and away from major vessels—ultrasound-guided percutaneous biopsy was considered feasible and safe. The multidisciplinary team therefore proceeded with ultrasound-guided liver biopsy to (i) establish a definitive diagnosis and (ii) reassess molecular markers in the metastatic focus to inform treatment planning. Histopathology demonstrated adenocarcinoma within liver tissue; in the context of the patient’s breast cancer history, metastatic disease was favored. Immunohistochemistry showed ER negative, PR negative, HER2 2+ (IHC), Ki-67 approximately 40%, GATA3 positive, E-cadherin positive, CK5/6 positive, and p40 negative. Fluorescence *in situ* hybridization (FISH) confirmed absence of HER2 gene amplification.

The discrepancy in HER2 IHC results between the primary tumor (HER2 1+) and the liver metastasis (HER2 2+ without amplification) was interpreted as reflecting intratumoral and inter-lesional heterogeneity. Receptor status—including hormone receptors and HER2—may change during disease progression from primary to metastatic sites, and prior studies have reported HER2 discordance rates of approximately 3%–16% between primary and metastatic lesions (8).

### 2.3 Local interventional treatment procedure

Based on imaging and ultrasound-guided biopsy, liver metastasis from triple-negative breast cancer was confirmed. An MDT comprising breast surgery, hepatobiliary surgery, and interventional radiology was convened to formulate the subsequent treatment plan. The therapeutic objective was to prioritize rapid intrahepatic tumor debulking and locoregional control within a systemic-therapy–based strategy, thereby creating a more durable disease-control window for ongoing systemic management. Given the abnormal liver function tests at the time of metastatic diagnosis, prompt reduction of hepatic tumor burden was considered clinically important to facilitate recovery of organ function. The MDT therefore favored a locoregional approach with relatively rapid onset that could be integrated seamlessly with systemic therapy.

Transarterial therapies represent an established category of locoregional options for the management of liver metastases. In TNBC-related breast cancer liver metastases, the clinical evidence largely comes from retrospective cohorts and systematic reviews evaluating modalities such as TACE and TARE. Collectively, these data indicate that, in carefully selected patients with liver-dominant involvement, transarterial approaches have an acceptable safety profile and can provide meaningful radiologic responses and intrahepatic disease contro^l^ (5) (9).

Within the overarching paradigm of transarterial locoregional therapy, the procedural choice in this case was individualized after balancing efficacy and toxicity. The CSCO breast cancer guidelines, in the section addressing adjuvant management of TNBC, recommend consideration of a carboplatin-containing regimen (e.g., paclitaxel plus carboplatin [TP]) for patients with residual disease or high-risk features such as nodal positivity (10). Accordingly, concomitant TP systemic chemotherapy was planned to enhance the likelihood of an objective response. Importantly, the patient had previously experienced grade 3 myelosuppression during radiotherapy, underscoring the need for careful control of treatment-related hematologic toxicity.

Against this background, the MDT favored bland transarterial embolization (TAE) rather than chemoembolization as the locoregional strategy for hepatic control. The intended therapeutic mechanism was superselective occlusion of tumor-feeding arteries to induce ischemic necrosis and rapidly reduce intrahepatic tumor burden, while minimizing the risk of compounded toxicity that could arise from combining intra-arterial chemotherapy with concurrent systemic chemotherapy (11).

Following MDT discussion, the team individualized the locoregional strategy for this patient with solitary liver metastasis and impaired hepatic function. Surgical resection, radiofrequency ablation, and transarterial approaches were considered. RFA was considered but not selected for two reasons. First, the metastatic lesion was located in hepatic segment V adjacent to the gallbladder fossa, and published literature identifies gallbladder proximity as a relative contraindication for percutaneous RFA due to the risk of thermal injury causing iatrogenic cholecystitis or gallbladder perforation (12). Second, the tumor size (3.0 × 2.7 cm) was at the upper boundary of optimal RFA efficacy; evidence demonstrates that RFA achieves the best local control for lesions <3 cm, with significantly higher recurrence and inferior disease-free survival for larger tumors (13).TARE was not selected because: (a) TARE is predominantly studied in the chemorefractory or salvage setting, whereas this patient was treatment-naïve for metastatic disease; (b) for a solitary, well-vascularized lesion, bland TAE was sufficient to achieve complete devascularization; and (c) TARE involves higher procedural cost and logistical complexity not warranted for a single lesion amenable to conventional embolization (5).Immunotherapy (e.g., pembrolizumab for PD-L1-positive TNBC) was discussed by the MDT. PD-L1 testing was not performed because the MDT determined it would not alter the treatment strategy: the patient presented with significant hepatic tumor burden requiring rapid cytoreduction; platinum-based chemotherapy achieves higher objective response rates (ORR) than immunotherapy in the first-line mTNBC setting; and immunotherapy has a slower onset of action, which was considered suboptimal given the clinical urgency. Antibody-drug conjugates (ADCs) such as sacituzumab govitecan were not yet widely available at the time of treatment (2021).Given the need for rapid cytoreduction to facilitate recovery of liver function and the desire to avoid delays related to surgical recovery, a minimally invasive approach that could be integrated with systemic chemotherapy was prioritized; therefore, superselective transarterial embolization (TAE) was selected.

After confirming adequate coagulation status, assessing hepatic and renal function, and excluding contraindications to endovascular therapy, digital subtraction angiography (DSA) was performed to characterize tumor vascularity and angiographic tumor staining. Superselective catheterization of tumor-feeding arteries was pursued to maximize on-target embolization and minimize ischemic injury to uninvolved liver parenchyma. Embolization was performed primarily using lipiodol to facilitate targeted deposition and post-procedural radiologic visualization, followed by adjunct particulate embolic agents (e.g., gelatin sponge particles and/or microspheres) as needed. The embolization endpoint was defined angiographically, based on marked flow stasis/slow flow and substantial reduction or disappearance of tumor blush. If residual micro-feeding branches or early reperfusion was suspected after the initial session, a short-interval imaging reassessment would be performed, and a planned consolidative superselective embolization could be considered to enhance devascularization and intrahepatic control.

The first TAE was performed on May 11, 2021. Superselective catheterization was achieved using a Renegade HI-FLO microcatheter. Embolization was performed using 10 mL of iodized oil (lipiodol) emulsified with 2 mL of normal saline (total 12 mL emulsion), of which 4 mL was delivered via the microcatheter until blood flow decelerated. This was followed by polyvinyl alcohol (PVA) microspheres (100–300 μm) and gelatin sponge particles. Periprocedural pain was managed with intramuscular pethidine (75 mg). No prophylactic antibiotics were administered, and no post-embolization syndrome or other significant periprocedural complications occurred. After the first TAE, although immediate angiography showed a significant reduction in tumor blush, microscopic feeding branches could still have been present. Therefore, the consolidative second TAE was performed on June 29, 2021 (7-week interval).

### 2.4 Systemic therapy and maintenance treatment

For systemic management, a TP regimen (paclitaxel plus carboplatin) was selected. Paclitaxel (400 mg) and carboplatin (600 mg) were administered every 3 weeks for 6 cycles. The primary treatment-related adverse event was grade III myelosuppression (CTCAE v5.0). Treatment response was evaluated according to RECIST 1.1 criteria. Following partial response, oral capecitabine maintenance (1,000 mg twice daily) was initiated on September 25, 2021 and continued for approximately 12 months (until September 2022).

At the time liver metastasis was confirmed, abnormal liver function tests suggested an urgent need for rapid cytoreduction to facilitate recovery of organ function; therefore, a combination regimen with relatively prompt antitumor activity was prioritized. Consistent with recommendations from major guidelines (CSCO, NCCN, and ESMO), platinum-containing regimens (including paclitaxel/carboplatin) may be considered in TNBC, particularly in patients with high-risk features such as nodal involvement and/or residual disease and without prior exposure to platinum in earlier treatment phases. Moreover, randomized studies in TNBC (e.g., GeparSixto (14) and CALGB 40603 (15)) have shown that adding carboplatin increases pathological response rates, with downstream implications for long-term outcomes. Although the patient had previously received taxane-based adjuvant therapy, the recurrence-free interval was ~14 months; given the clinical context at relapse, re-introduction of a taxane in combination with carboplatin was considered reasonable to enhance the likelihood of objective response. Although the patient had previously received a taxane-based adjuvant regimen, the recurrence-free interval was ~14 months; in the context of metastatic relapse, re-introduction of a taxane in combination with carboplatin was considered reasonable to maximize the probability of objective response. Given the visceral metastatic setting of TNBC, even after a substantial response to combined TAE and TP, consolidation during the disease-control phase was deemed important to prolong the progression-free interval. The international Advanced Breast Cancer (ABC) consensus states that, in metastatic breast cancer, maintenance treatment after achieving clinical benefit is typically continued until disease progression, unacceptable toxicity, or patient preference, and should be individualized through careful balancing of efficacy and cumulative toxicity (16) (17). In addition, real-world data in metastatic TNBC (mTNBC) indicate that, among patients who achieve disease control on first-line platinum-based therapy, continuation with maintenance chemotherapy may be associated with improved outcomes, including longer PFS and OS (e.g., PFS 9.63 vs 7.47 months; OS 31.27 vs 25.37 months (18)).Capecitabine, an oral agent with a generally favorable tolerability profile, has also been reported as a maintenance option that may reduce the risk of severe myelosuppression and support longer-term outpatient management (19). Accordingly, following multidisciplinary assessment of anticipated benefit and tolerability, the patient was prescribed approximately 1 year of oral capecitabine maintenance to consolidate systemic control while minimizing treatment burden and aiming to reduce the risk of disease reactivation.

### 2.5 Follow-up and outcomes

**Figure 3:**
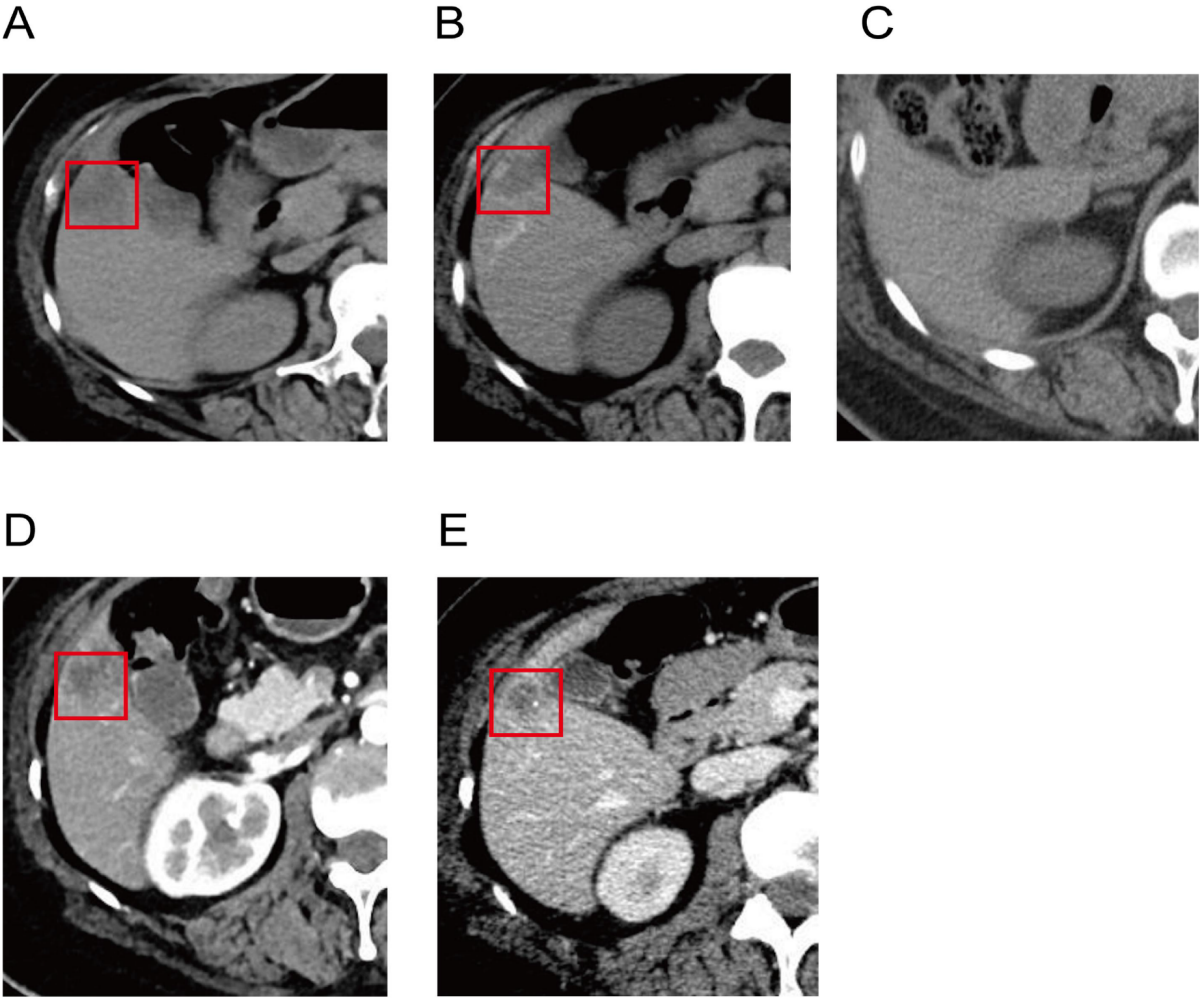
Serial abdominal CT demonstrating treatment response and tumor devascularization. Upper row: Non-contrast CT. **(A)** Pre-TAE (May 2, 2021): Hypodense lesion in hepatic segment V (3.0 × 2.7 cm, red box) with central necrosis. **(B)** Approximately 7 weeks after first TAE (June 28, 2021): Lesion decreased to 2.2 × 1.5 cm (red box) with lipiodol deposition. **(C)** Approximately 5 months after second TAE (November 10, 2021): Further regression to 1.7 × 1.2 cm. Lower row: Contrast-enhanced CT. **(D)** Pre-TAE (May 2, 2021): Hepatic segment V lesion (red box) with peripheral rim enhancement, indicating arterial blood supply. **(E)** Approximately 7 weeks after first TAE (June 28, 2021): Marked reduction in arterial enhancement (red box), consistent with successful tumor devascularization following embolization.

**Figure 4:**
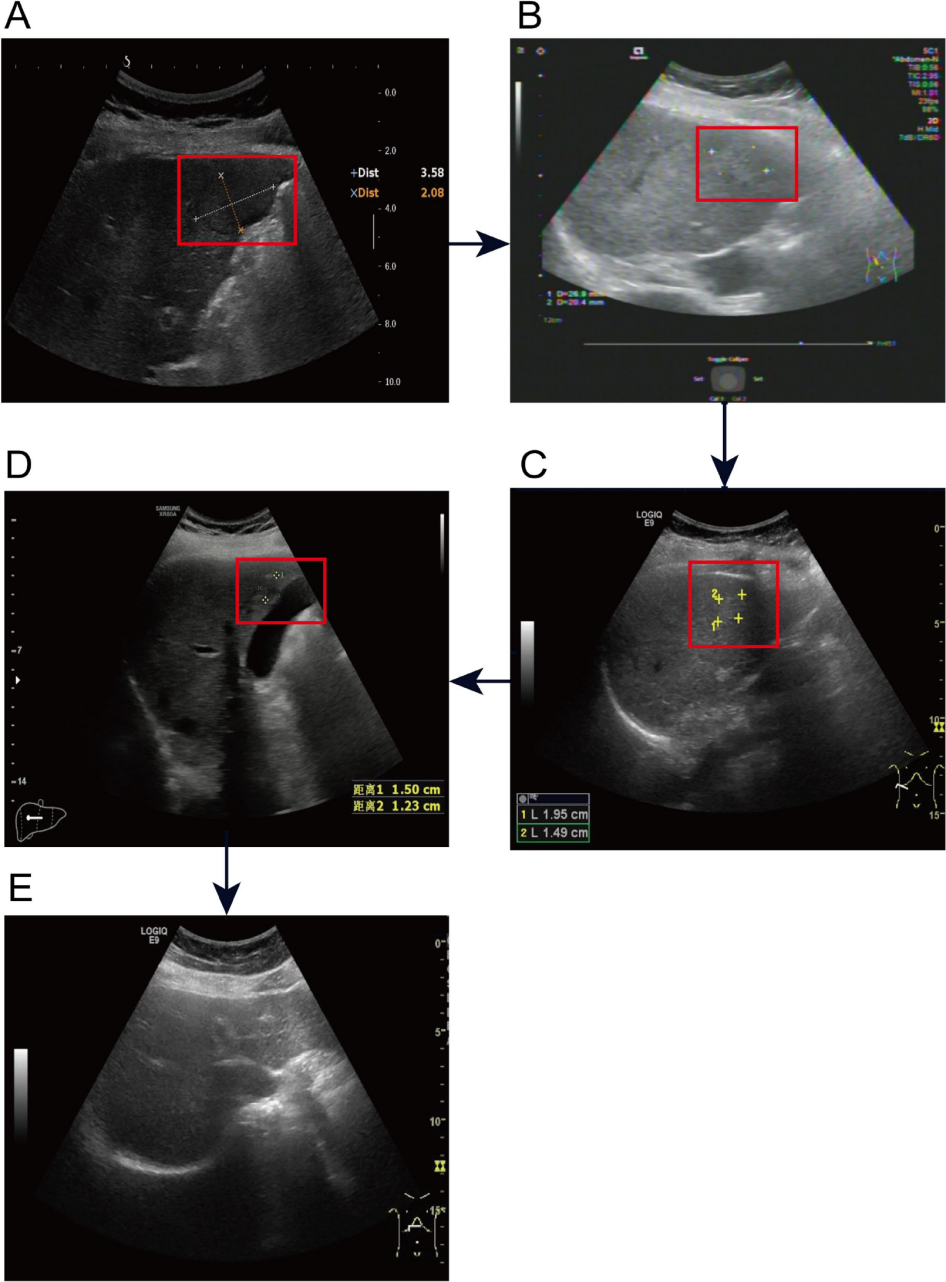
Serial liver ultrasonography demonstrating progressive regression of hepatic metastasis to complete resolution. **(A)** Baseline, pre-TAE (May 2, 2021): Hypoechoic nodule (3.58 × 2.08 cm) in hepatic segment V near the gallbladder fossa (red box). **(B)** Approximately 3 weeks after first TAE (June 4, 2021): Lesion decreased to approximately 2.7 × 2.0 cm (red box), indicating early treatment response. **(C)** Approximately 5 weeks after second TAE (August 5, 2021): Further regression to 1.95 × 1.49 cm (red box). **(D)** Approximately 8 months after second TAE (February 10, 2022): Continued regression to 1.50 × 1.23 cm (red box). **(E)** Approximately 19 months after second TAE (February15, 2023): No focal hepatic lesion detectable, consistent with complete radiologic regression. Arrows indicate the chronological sequence of imaging follow-up.

**Figure 5:**
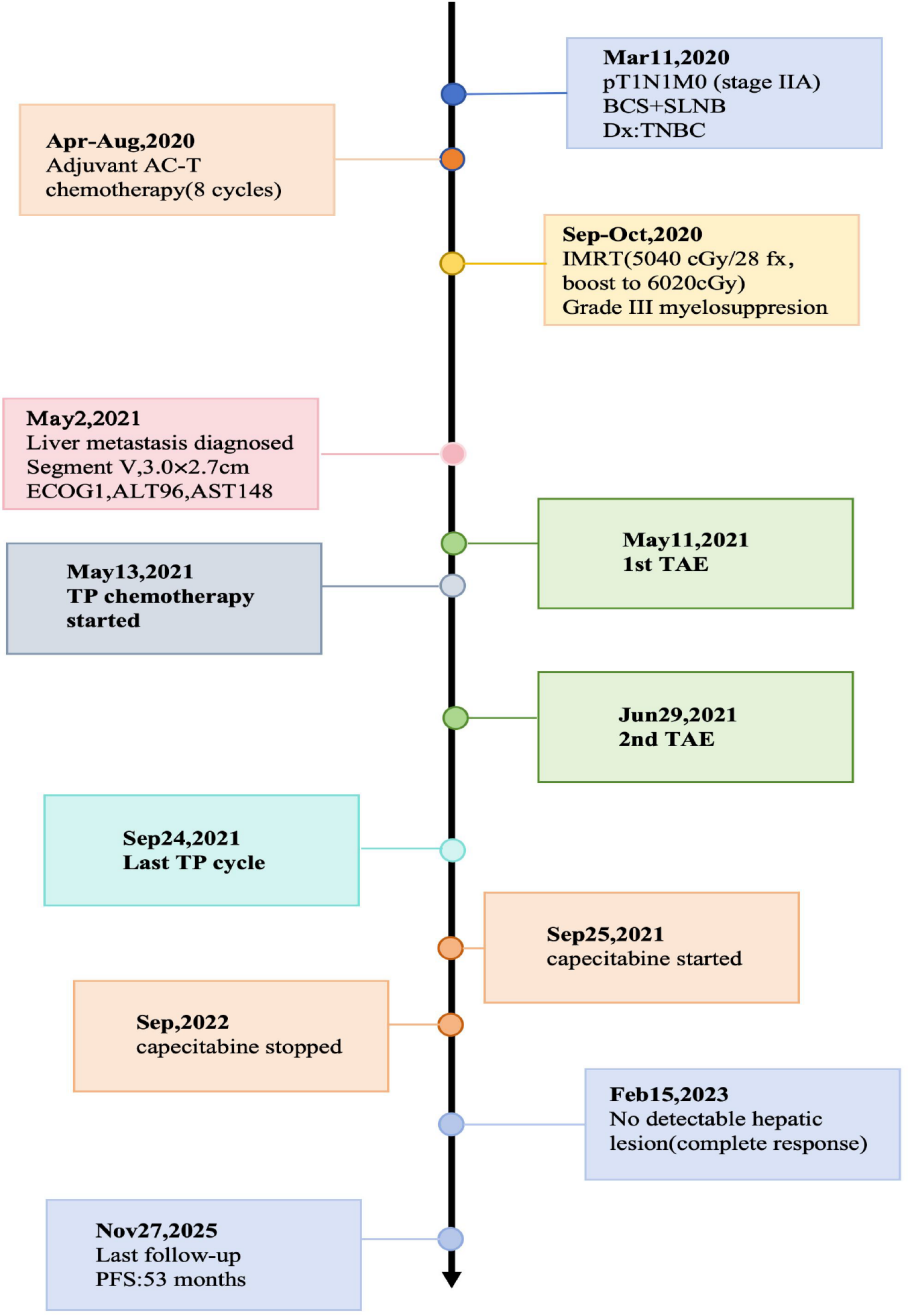
Clinical timeline of disease course, treatment, and follow-up.

Following TAE in combination with a TP regimen (paclitaxel plus carboplatin), follow-up imaging demonstrated a partial response (**Figures 3**, **4**). At the last follow-up on November 27, 2025, the patient had achieved a progression-free survival of 53 months as summarized in the clinical timeline (**Figure 5**). Surveillance imaging initially included non-contrast abdominal CT and abdominal ultrasonography every 3–6 months, which was subsequently extended to annual assessments. Across all follow-up evaluations, there was no radiologic evidence of recurrent or metastatic disease. The most recent non-contrast abdominal CT on November 27, 2025, continued to demonstrate no signs of disease recurrence.

Serial tumor markers, including CA15–3 and CEA, remained within normal limits during follow-up. Liver function tests improved gradually after locoregional intervention combined with systemic therapy and remained stable thereafter, without clinically meaningful fluctuations across the surveillance period. At present, the patient remains in good general condition with preserved quality of life. No clinically significant treatment-related adverse events have been reported, and her activities of daily living and work capacity remain intact.

## 3 Discussion and conclusion

Liver metastasis in triple-negative breast cancer (TNBC) is associated with a dismal prognosis. Prior reports describe a median survival of approximately 3–15 months, and even with contemporary therapies, the 5-year survival rate remains below 12% (20).In contrast to these historical outcomes, our patient with TNBC liver metastasis achieved a progression-free survival exceeding four years following transarterial embolization combined with systemic chemotherapy and ultimately attained complete radiologic remission, representing an unusually durable clinical course relative to published expectations.

To our knowledge, this represents the longest PFS reported following bland TAE specifically for TNBC liver metastasis. Published systematic reviews report median OS of 15.3 months for TACE and 11.9 months for TARE across mixed breast cancer subtypes (5), with pooled median OS of 17.8 months for TACE and 9.2 months for TARE (7). The longest previously reported median OS was 47 months in a mixed-subtype cohort receiving TACE combined with systemic chemotherapy. Our case is unique in combining: (a) the TNBC subtype, which carries the worst prognosis among BCLM; (b) use of bland TAE rather than TACE or TARE, minimizing toxicity overlap; (c) PFS of 53 months with complete radiologic remission; and (d) successful de-escalation to capecitabine maintenance.

Locoregional interventional approaches have become an important component of management for breast cancer liver metastases. Although hepatic resection can offer the possibility of long-term disease control in highly selected patients, eligibility for curative-intent surgery is limited (21), particularly in those with high hepatic tumor burden or after failure of systemic therapy. Accordingly, locoregional interventional therapies have emerged as important adjuncts in this setting. Published reviews describe palliative locoregional options for breast cancer liver metastases, including bland TAE, TACE, transarterial radioembolization/selective internal radiation therapy (SIRT), and thermal ablation modalities such as radiofrequency or microwave ablation (21). TAE achieves locoregional tumor control by selectively occluding tumor-feeding arteries with embolic materials, thereby inducing ischemia and subsequent tumor necrosis (22).In contrast, TACE adds chemotherapeutic agents on the basis of embolization to enhance efficacy. Prior studies have reported that, among patients with breast cancer liver metastases, TACE is associated with longer survival compared with systemic chemotherapy alone (23). Xu et al. reported a cohort of 14 patients who underwent DEB-TACE. The objective response rate at 3 months was 71.4%, and the 6-month disease control rate was 71.4%; nevertheless, the median local progression-free survival was only 8.0 months (24). A 2025 systematic review likewise suggested that TACE and TARE represent effective locoregional options. Although the evidence base is largely retrospective, the available data indicate encouraging overall survival and disease control in appropriately selected patients (5). Importantly, TAE/TACE and other locoregional modalities have complementary strengths and limitations. Hepatic resection is invasive and feasible only in a highly selected minority (21) whereas thermal ablation is typically best suited for small, isolated lesions. By contrast, Yttrium-90 radioembolization (TARE) has a distinct toxicity profile and is often considered in multifocal disease or in chemotherapy-refractory settings (5). In the present case, a strategy combining TAE with systemic chemotherapy was selected to leverage its potential for effective intrahepatic tumor control while maintaining compatibility with concurrent systemic treatment.

Effective intrahepatic tumor control is critical for preserving hepatic function and extending the opportunity for ongoing systemic therapy. Prior studies suggest that, in patients with liver-dominant disease and relatively stable extrahepatic burden, hepatic arterial interventions may slow intrahepatic progression and help delay liver function decline (22). By debulking intrahepatic tumor burden, locoregional interventional therapy may reduce the risk of hepatic decompensation and allow patients to remain eligible for systemic chemotherapy or other systemic options. This consideration is especially relevant in TNBC, where systemic treatment choices are comparatively limited and clinical progression can be rapid. Achieving intrahepatic disease control may mitigate liver-related complications and reduce hepatic tumor burden, thereby helping preserve treatment tolerance and enabling sustained anticancer therapy. For instance, some reports recommend integrating hepatic locoregional intervention during breaks or intervals in systemic therapy to preserve liver function and potentially extend overall treatment benefit (22). In this case, TAE was followed by durable intrahepatic disease control and a progression-free interval exceeding four years, highlighting the potential value of effective locoregional control in extending the overall therapeutic window and preserving liver function.

In terms of safety, recent systematic reviews indicate that TACE and TARE have an acceptable tolerability profile, with relatively low rates of grade 3 or higher adverse events/complications (5). Collectively, available studies suggest that TACE/TARE may serve as palliative locoregional options for patients with liver-dominant breast cancer, with relatively low rates of high-grade adverse events in appropriately selected cohorts. In the present case, no irreversible hepatic dysfunction was observed during TAE combined with chemotherapy. Liver function remained well preserved, allowing uninterrupted continuation of subsequent systemic therapy. In summary, locoregional hepatic interventions—particularly transarterial embolization–based approaches (bland embolization and/or chemoembolization)—may represent a valuable adjunct to systemic therapy for selected patients with TNBC liver metastases. The aims of such approaches are to achieve effective intrahepatic tumor control, preserve hepatic function, and thereby extend the overall opportunity for sustained anticancer therapy. Although high-level evidence from large prospective studies remains limited, the existing real-world experience and retrospective analyses generally support the feasibility, safety, and potential efficacy of these approaches (24). This case suggests that, following multidisciplinary evaluation, integrating locoregional interventional therapy into a multimodal, sequential treatment strategy may contribute to durable disease control in selected patients with TNBC liver metastases, offering practical insights for future individualized management.

Regarding emerging therapeutic options, immunotherapy with checkpoint inhibitors (e.g., pembrolizumab in combination with chemotherapy for PD-L1-positive mTNBC) and antibody-drug conjugates (e.g., sacituzumab govitecan) have transformed the treatment landscape for mTNBC. In our case (2021), the MDT prioritized rapid cytoreduction given significant hepatic tumor burden, and platinum-based chemotherapy was selected for its higher ORR and faster onset compared with immunotherapy. ADCs were not yet widely accessible. Future cases presenting in the current therapeutic era should incorporate these agents into MDT discussions, potentially in combination with locoregional interventions.

This case suggests that in selected patients with isolated liver metastasis from TNBC, TAE combined with systemic chemotherapy may achieve long-term tumor control. By rapidly reducing tumor burden and enhancing local control, TAE potentially extends the window for effective systemic therapy. Given that TNBC liver metastasis typically carries a poor prognosis with median PFS often limited to a few months (25), the PFS of over 53 months observed in this patient significantly exceeds expectations and highlights the potential clinical benefit of this combined approach. The patient demonstrated excellent tolerance and maintained a high quality of life throughout treatment, indicating good safety and feasibility. This case provides valuable insight into individualized treatment planning for TNBC liver metastasis and supports further exploration of incorporating locoregional interventions such as TAE into multidisciplinary treatment strategies.

Several limitations should be acknowledged. First, as a single case report, a causal relationship between TAE and prolonged PFS cannot be established; the favorable outcome may reflect a combination of locoregional intervention, effective systemic therapy, and individual tumor biology. Second, this patient had features associated with better prognosis, including solitary metastasis (oligometastatic disease), good performance status (ECOG 1), absence of extrahepatic disease, and preserved hepatic function despite elevated transaminases, which limit generalizability. Third, although tumor heterogeneity was confirmed (HER2 discordance between primary and metastatic sites), indolent tumor biology cannot be excluded as a contributing factor. Fourth, PD-L1 and BRCA testing were not performed, representing additional limitations in molecular characterization. Prospective studies are warranted to validate the role of bland TAE combined with systemic chemotherapy in oligometastatic TNBC and to identify optimal patient selection criteria.

## Data Availability

The original contributions presented in the study are included in the article/supplementary material. Further inquiries can be directed to the corresponding author.

## Glossary

ADC: Antibody-drug conjugate;
BI-RADS: Breast Imaging Reporting and Data System;
CTCAE: Common Terminology Criteria for Adverse Events;
CK5/6: Cytokeratin 5/6;
CSCO: Chinese Society of Clinical Oncology;
CT: Computed tomography;
DEB-TACE: Drug-eluting bead transarterial chemoembolization;
DSA: Digital subtraction angiography;
ECOG: Eastern Cooperative Oncology Group;
ER: Estrogen receptor;
ESMO: European Society for Medical Oncology;
FISH: Fluorescence *in situ* hybridization;
GATA3: GATA binding protein 3;
HER2: Human epidermal growth factor receptor 2;
IHC: Immunohistochemistry;
IMRT: Intensity-modulated radiotherapy;
MDT: Multidisciplinary team;
MRI: Magnetic resonance imaging;
mTNBC: Metastatic triple-negative breast cancer;
NCCN: National Comprehensive Cancer Network;
NST: No special type;
OS: Overall survival;
ORR: Objective response rate;
PVA: Polyvinyl alcohol;
PFS: Progression-free survival;
PR: Progesterone receptor;
RECIST: Response Evaluation Criteria in Solid Tumors;
RFA: Radiofrequency ablation;
SIRT: Selective internal radiation therapy;
TAE: Transarterial embolization;
TACE: Transarterial chemoembolization;
TARE: Transarterial radioembolization;
TNBC: Triple-negative breast cancer;
TP: Paclitaxel and carboplatin.
